# Unplanned Readmission in Outpatient Hand Surgery: An Analysis of 23,613 Patients in the NSQIP Data Set

**Published:** 2017-12-06

**Authors:** Daniel P. Donato, Alvin C. Kwok, Michael O. Bishop, Angela P. Presson, Jayant P. Agarwal

**Affiliations:** ^a^Division of Plastic Surgery; ^b^Division of Epidemiology, Study Design and Biostatistics Center, University of Utah School of Medicine, Salt Lake City

**Keywords:** hand surgery, readmission, complications, pain control, NSQIP

## Abstract

**Objective:** In an era of controlling cost and improving care, 30-day readmission rates have become an important quality measure. The purpose of this study was to identify the rates of 30-day unplanned readmission and the associated risk factors in patients undergoing outpatient hand surgery. **Methods:** The 2011-2014 National Surgical Quality Improvement Project data were queried for patients who met 368 hand-specific Current Procedural Terminology codes. Univariable and multivariable analyses were performed to identify patient- and surgery-specific risk factors associated with unplanned readmission within 30 days. **Results:** Of the 368 Current Procedural Terminology codes queried, 208 were represented in the data, for a total of 23,613 patients. The overall unplanned readmission rate was 0.88% (207/23,613). On both univariable and multivariable analyses, operative year (2012), increasing age, obesity, smoking status, chronic obstructive pulmonary disease, preoperative steroid use, preoperative anemia, increasing American Society of Anesthesiologists classification, increasing operative time, and a procedure performed by a surgeon other than a plastic or orthopedic surgeon were associated with increased readmission rates. Diabetes, hypertension, low albumin levels, elevated international normalized ratio, and dirty/infected wound classification were only significant in univariable analysis. Current Procedural Terminology codes associated with the highest readmission rates were related to amputations. The most common readmission diagnoses were wound complications, followed by uncontrolled postoperative pain. **Conclusions:** The incidence of unplanned readmission is low in patients undergoing outpatient hand surgery. Specific patient comorbidities are associated with increased unplanned readmission rates. This information may be useful in identifying patients at higher risk for unplanned readmission and in counseling of high-risk patients preparing for surgery.

In an effort to control cost and improve care, 30-day readmission rates have become an important quality measure. Hospital readmissions in both the United States and Europe are associated with increased cost. National programs such as the Centers for Medicare & Medicaid Services Hospital Readmissions Reduction Program have been implemented to reduce hospital readmissions by imposing payment penalties for hospitals with above-average readmission rates.^1^ A Medicare Payment Advisory Commission analysis found that unplanned readmissions in the United States accounted for a $15 billion annual expenditure, representing 17.6% of Medicare costs.^2^ An estimated $12 billion of this expenditure was potentially preventable.^2^ Hospital readmissions after surgery result in higher costs for patients and an increase in overall health care expenditure.^1^^,^^3^ Recent large studies have shown that complications in hand surgery are relatively low, occurring in 2.5% of cases.^4^

Most hand surgeries procedures are performed in an outpatient setting. Complications requiring an unplanned readmission can result in significant morbidity and cost. While readmission data have been studied in other surgical fields to identify baseline rates and risk factors, little is known about the readmission rates after outpatient hand surgery.^5^^-^11

The purpose of this study was to perform a retrospective analysis of the National Surgical Quality Improvement Project (NSQIP) database to determine the rates of 30-day unplanned readmission in patients undergoing outpatient hand surgery. The NSQIP provides a validated, prospectively collected database of patients undergoing varying surgical procedures at more than 760 institutions. We also sought to identify patient characteristics and comorbidities, as well as perioperative risk factors for readmission.

## METHODS

### Study design

A retrospective study was conducted using the NSQIP database for patients undergoing outpatient hand surgery from the years 2011 through 2014. The history and methods of the NSQIP database have been described in detail previously.^12^^-^14

The NSQIP participant data files were obtained from 2011 through 2014. The database was queried for 368 hand-specific Current Procedural Terminology (CPT) codes. We excluded primary CPT codes that could correspond to procedures performed on other areas of the body. We then selected for patients whose hand procedure was performed in the outpatient setting.

We identified patient and perioperative factors that were associated with higher 30-day readmission rates after outpatient hand surgery. Variables selected for analysis included patient demographics (age, gender, obesity, and race), comorbidities (diabetes, smoking status, chronic obstructive pulmonary disease [COPD], hypertension, preoperative steroid use, laboratory data, and American Society of Anesthesiologists [ASA] classification), and perioperative factors (emergency surgery, surgical wound classification, and operative time). Obesity was defined as a body mass index of greater than 35 kg/m^2^. Laboratory data included creatinine, hematocrit, and international normalized ratio (INR). Patient laboratory results were assessed for abnormalities, and their results were listed (listed as “missing” if laboratory values were not drawn). Anemia was defined as a hematocrit of less than 28%. ASA classification is a widely used marker of overall health and was assigned by the anesthesiologist at the time of surgery.

Our primary outcome was unplanned readmission within 30 days of the index surgery. Secondary outcomes were defined as wound complications (superficial infection, wound infection, deep or organ space infection, wound dehiscence, or graft or flap failure), medical complications (myocardial infarction, pneumonia, unplanned intubation, urinary tract infection, stroke, pulmonary embolism, deep vein thrombosis, renal insufficiency, sepsis, and death), bleeding, and reoperation within 30 days.

### Statistical analysis

Patient characteristics and perioperative factors were summarized as median and interquartile range for age and operative time, and count (%) for categorical variables, stratified by unplanned readmission within 30 days. There were high rates of missing laboratory data (55%-88%) likely due to tests not being ordered and thus the absence of a test result was coded as its own level for analysis (levels included normal, abnormal, missing/not ordered). Race, with 22% missing, was handled similarly. Other variables had low rates of missing values (<2%), and casewise deletion was used for their analysis. Univariable and multivariable logistic regressions were used to analyze primary (unplanned readmission within 30 days) and secondary outcomes (wound complications, medical complications, bleeding, and reoperation within 30 days). Odds ratios (ORs) with their associated 95% confidence intervals (CIs) and *P* values were reported from these models. We also reported the top procedures with the highest readmission rates (# of readmissions for procedure *i*/# of patients with procedure *i*). Procedures that only had 1 occurrence of patient readmission were excluded from subgroup analysis. Analyses were performed using R, version 3.3.1.^15^

## RESULTS

Of the 368 hand-specific CPT codes queried, 208 distinct CPT codes were identified in the database, representing a total of 23,613 patients who underwent outpatient hand surgery. There was nearly an even distribution between men and women, 46% and 54%, respectively. Sixty-seven percent of patients were white, and 81% of patients had an ASA class of either 1 or 2. Fifty-five percent of patients had no preoperative laboratory values drawn in the 30 days prior to surgery. Most procedures were performed by plastic or orthopedic surgeons (96%) under general anesthesia (68%) involving clean surgical wounds (93%) (Table 1).

Among the patients who underwent outpatient hand surgery, 207 (0.88%) had an unplanned readmission within 30 days of their index procedure. Of these, 31% had a complication, where 18% had a wound complication and 14% had a medical complication (Table 2). One patient was readmitted for both medical and surgical complications. Among the readmissions, 33% had a reoperation. In comparison, only about 1% of patients who were not readmitted had a complication or reoperation.

### Univariable analysis

On univariable analysis, individual patient characteristics associated with unplanned readmission included the operative year (2012), increasing age, obesity, diabetes, smoking status, COPD, hypertension, preoperative steroid use, and increasing ASA classification (Table 1). Surgeon specialty other than plastic or orthopedic surgery, longer operative times, and dirty/infected wound classification were all variables that also showed statistically significant associations with unplanned readmission. Patients who had preoperative laboratory values drawn for anemia, creatinine, or INR within 30 days of surgery, regardless of whether there were any abnormalities, had increased unplanned readmission rates. Increased creatinine levels and preoperative anemia showed an association with an increased risk of unplanned readmission, whereas the presence of an elevated INR did not. No association was found between unplanned readmission and gender, race, type of anesthesia used, emergency surgery, or other procedures performed concurrently (Table 1).

### Multivariable analysis

In a multivariable model, factors independently associated with unplanned readmission included, operative year (2012) (OR = 1.90; 95% CI = 1.14-3.30), increasing age (OR = 1.06, 95% CI = 1.01-1.12), obesity (OR = 1.45, 95% CI = 1.01-2.06), smoking status (OR = 1.69, 95% CI = 1.21-2.33), COPD (OR = 1.88, 95% CI = 1.09-3.07), preoperative steroid use (OR = 1.89, 95% CI = 1.00-3.28), preoperative anemia (OR = 3.74, 95% CI = 1.29-9.29), ASA class 2 (OR = 1.93, 95% CI = 1.14-3.46), ASA class 3 (OR = 2.50, 95% CI = 1.34-4.84), ASA class 4 (OR = 3.33, 95% CI = 1.24-8.54), increasing operative time (OR = 1.04, 95% CI = 1.00-1.07), and a procedure performed by a specialty other than a plastic or orthopedic surgeon (OR = 1.82, 95% CI = 1.03-3.02). Diabetes, hypertension, an absence of laboratory data, or dirty/infected wound classification, despite significance in univariable analysis, showed no association with increased unplanned readmission rates in the multivariable model (Table 1).

### Subgroup analysis

On examining the patients who underwent unplanned readmission, the CPT codes with the highest readmission rates were related to amputations (Table 3). Metacarpal amputation readmission rate was 12.5%, and finger amputation was 3.57% with direct closure and 3.26% with flap closure. Other procedures associated with higher readmission rates included percutaneous pinning of Bennett's fractures, open reduction and internal fixation (ORIF) of proximal or middle phalanx fractures, repair of zone 2 flexor tendon injuries, and ORIF of comminuted distal radius fractures.

Of those patients with a known readmission diagnosis, the most frequent reason for readmission was related to wound infections or wound dehiscence (42%), followed by uncontrolled postoperative pain (16%) and medical complications (13%) (Table 4). Emergent complications such as bleeding requiring transfusion and compartment syndrome were rare occurrences, with 2% and 1%, respectively. Wound complications occurred at an average of 15 days after the index procedure. Readmission due to uncontrolled postoperative pain occurred at an average of 3.46 days after surgery; however, the majority occurred within 1 day postoperatively.

## DISCUSSION

In the current health care climate, complications and unplanned readmissions are critical measures of quality. Readmission is an unwanted and costly outcome, and analyzing large-scale outcome-based databases allows for the identification of patient- and surgery-specific risk factors. High-risk patients could be identified and targeted in hopes of driving down readmission rates and their associated costs and complications.

Overall complication rates for hand surgery have previously been shown to be much lower than other surgical areas.^4^ In our study of 23,613 outpatient hand surgical procedures, the overall unplanned readmission rate was low, at 0.88%. This is much lower than the unplanned readmission rates seen in other specialties when performed as an outpatient procedure, which range between 1.2% and 6.3%.^7^^,^^16^^,^^17^ Patients undergoing outpatient surgery tend to be healthier, and procedures tend to be shorter and less invasive. While outpatient hand surgery has a low rate of readmission, a readmission can nonetheless result in significant morbidity and cost. Understanding the reasons for readmission can help a surgeon avoid this complication.

Patients undergoing amputations, especially at the metacarpal level, were at a significantly increased risk of having an unplanned readmission. The increased readmission risk in these patients makes it reasonable that patients be carefully screened prior to undergoing these procedures on an outpatient basis. Those with significant comorbidities such as obesity, diabetes, and steroid use may be more suited to having these procedures done with an overnight inpatient stay or 23-hour observation status.

The most frequent reasons for unplanned readmission are related to wound infections and wound complications. These total almost half of all unplanned readmissions. Readmission occurs on average 15.9 days after surgery. Frequent, planned follow-up with either the surgical team or hand therapists may allow for early identification and outpatient treatment of wound-related complications. More frequent follow-ups are inevitably cheaper than an unplanned readmission. In addition, patient education focused on early signs of wound deterioration may prevent unplanned readmission.

We found that 14% of patients with a known readmission diagnosis in outpatient hand surgery carried a primary diagnosis of uncontrolled postoperative pain. Previous studies have shown that pain alone can be a significant factor in patients seeking care after surgery.^18^^-^20 Menendez and Ring^20^ found that 16% of emergency department visits after hand surgery at their institution were pain related, and Curtin and Hernandez-Boussard^18^ found that 10% of readmissions after distal radius fracture were related to pain. In our cohort, these patients were most commonly readmitted on the day of surgery or postoperative day 1. It is possible that this coincides with pain relief from local or regional anesthetic blocks. Developing effective postoperative pain management strategies after hand surgery procedures is critically important for patient comfort, to prevent readmission, and to allow for effective rehabilitation. This can include long-acting local/regional anesthetics, nonopioid adjuncts such as nonsteroidal anti-inflammatory drugs or γ-aminobutyric acid analogues, and patient education. Appropriate pain control in the immediate postoperative period can cut down on the need for emergency department visits and unplanned readmissions.

Data on carpal tunnel surgery are not collected in the NSQIP database. This is the most common hand surgery performed annually and is almost always performed on an outpatient basis.^21^ Carpal tunnel surgery is associated with very low complication and readmission rates.^22^^,^^23^ The lack of carpal tunnel surgery in our cohort may result in an overestimation of complication and readmission rates in outpatient hand surgery. A recent single-center study showed that 28,737 ambulatory hand surgical procedures had an overall complication rate of 0.2% and an unplanned readmission rate of less than 0.03% (8 of 28,737 cases). In our data, the lowest incidence of unplanned readmission did not approach these low rates, which may, in part, be related to overestimation due to lack of carpal tunnel data.

This study is a step toward identifying an important quality outcome and its relationship to outpatient hand surgery. These data can be used to identify patients who are at higher risk of unplanned readmission after outpatient hand surgery. It will also be helpful in preoperative counseling of patients preparing for surgery and in targeting further studies for higher risk patients and higher risk procedures performed in the outpatient setting.

## Figures and Tables

**Table 1:** Patient and surgery characteristics stratified by unplanned readmission in all outpatient hand surgery*

	Patient characteristics		ORs for readmission			
Patient characteristics	Unplanned readmission (N = 207)	No unplanned readmission (N = 23,406)	Univariable OR (95% CI)*	*P*	Multivariable OR (95% CI)*†	*P*
Admission year						
2011	20 (10%)	3257 (14%)	Reference	Ref.	Ref.	Ref.
2012	55 (27%)	4932 (21%)	1.82 (1.11-3.11)^‡^	.023	1.90 (1.14-3.30)^‡^	17.018
2013	61 (29%)	6502 (28%)	1.53 (0.94-2.60)	.10	1.56 (0.94-2.69)	.096
2014	71 (34%)	8715 (37%)	1.33 (0.82-2.24)	.27	1.32 (0.81-2.27)	.29
Age (5-y increments)	11.4 (9.3-13.6)	10 (6.6-12.4)	1.14 (1.09-1.18)^‡^	<.001	1.06 (1.01-1.12)^‡^	.033
Gender^‖^						
Female	120 (58%)	12,622 (54%)	Ref.	Ref.	Ref.	Ref.
Male	87 (42%)	10,781 (46%)	0.85 (0.64-1.12)	.25	1.05 (0.78-1.41)	.75
Race						
White	142 (69%)	15,578 (67%)	Ref.	Ref.	Ref.	Ref.
Other races	31 (15%)	2726 (12%)	1.25 (0.83-1.82)	.27	1.34 (0.88-1.98)	.15
Missing	34 (16%)	5102 (22%)	0.73 (0.49-1.05)	.10	1.09 (0.72-1.61)	.68
Obese						
No	161 (78%)	20,357 (87%)	Ref.	Ref.	Ref.	Ref.
Yes	46 (22%)	3049 (13%)	1.91 (1.36-2.63)^‡^	<.001	1.45 (1.01-2.06)^‡^	.041
Diabetes						
No	170 (82%)	21,651 (93%)	Ref.	Ref.	Ref.	Ref.
Yes	37 (18%)	1755 (7%)	2.69 (1.85-3.80)^‡^	<.001	1.34 (0.89-1.99)	.15
Smoker						
No	142 (69%)	18,039 (77%)	Ref.	Ref.	Ref.	Ref.
Yes	65 (31%)	5367 (23%)	1.54 (1.14-2.06)^‡^	.004	1.69 (1.21-2.33)^‡^	.002
COPD						
No	187 (90%)	22,855 (98%)	Ref.	Ref.	Ref.	Ref.
Yes	20 (10%)	551 (2%)	4.44 (2.69-6.91)^‡^	<.001	1.88 (1.09-3.07)^‡^	.016
Hypertension						
No	113 (55%)	17,334 (74%)	Ref.	Ref.	Ref.	Ref.
	94 (45%)	6072 (26%)	2.37 (1.80-3.12)^‡^	<.001	1.17 (0.83-1.65)	.36
Preoperative steroid use						
No	194 (94%)	22,931 (98%)	Ref.	Ref.	Ref.	Ref.
Yes	13 (6%)	475 (2%)	3.23 (1.74-5.49)^‡^	<.001	1.89 (1.00-3.28)^‡^	.036
Anemic						
Missing	76 (37%)	12,965 (55%)	Ref.	Ref.	Ref.	Ref.
No	125 (60%)	10,351 (44%)	2.06 (1.55-2.75)^‡^	<.001	0.95 (0.63-1.46)	.82
Yes	6 (3%)	90 (0%)	11.37 (4.33-24.76)^‡^	<.001	3.74 (1.29-9.29)^‡^	.008
ASA class^‖^						
1. No disturb	17 (8%)	6248 (27%)	Ref.	Ref.	Ref.	Ref.
2. Mild disturb	102 (50%)	12,594 (54%)	2.98 (1.83-5.15)^‡^	<.001	1.93 (1.14-3.46)^‡^	.020
3. Severe disturb	76 (37%)	4092 (18%)	6.83 (4.13-11.95)^‡^	<.001	2.50 (1.34-4.84)^‡^	.005
4. Life threat	10 (5%)	205 (1%)	17.93 (7.82-39.00)^‡^	<.001	3.33 (1.24-8.54)^‡^	.014
Low albumin						
Missing	141 (68%)	19,747 (84%)	Ref.	Ref.	…	…
No	66 (32%)	3648 (16%)	2.53 (1.88-3.39)	<.001	…	…
Yes	0 (0%)	11 (0%)	…^§^	>.999^§^	…	…
Elevated creatinine						
Missing	71 (34%)	13,626 (58%)	Ref.	Ref.	Ref.	Ref.
No	122 (59%)	9520 (41%)	2.46 (1.84-3.31)^‡^	<.001	1.51 (0.97-2.36)	.071
Yes	14 (7%)	260 (1%)	10.33 (5.53-18.01)^‡^	<.001	2.91 (1.30-6.19)^‡^	.007
Elevated INR						
Missing	162 (78%)	20,634 (88%)	Ref.	Ref.	Ref.	Ref.
No	43 (21%)	2705 (12%)	2.02 (1.43-2.81)^‡^	<.001	1.14 (0.78-1.66)	.49
Yes	2 (1%)	67 (0%)	3.80 (0.62-12.26)	.06	1.32 (0.21-4.60)	.71
Concurrent procedure						
No	206 (100%)	23,346 (100%)	Ref.	Ref.	Ref.	Ref.
Yes	1 (0%)	60 (0%)	1.89 (0.11-8.62)	.53	1.71 (0.10-8.03)	.60
Surgery specialty						
Orthopedics	147 (71%)	17,735 (76%)	Ref.	Ref.	Ref.	Ref.
Plastics	43 (21%)	4751 (20%)	1.09 (0.77-1.52)	.61	1.26 (0.87-1.79)	.22
Other	17 (8%)	920 (4%)	2.23 (1.30-3.59)^‡^	.002	1.82 (1.03-3.02)^‡^	.028
Operation time^‖^ (15-min groups)	4.3 (2.5-6.2)	3.7 (2.2-5.5)	1.04 (1.00-1.07)^‡^	.016	1.04 (1.00-1.07)^‡^	.033
Anesthesia^‖^						
General	148 (71%)	15,938 (68%)	Ref.	Ref.	Ref.	Ref.
Other	58 (28%)	7465 (32%)	0.84 (0.61-1.13)	.26	0.82 (0.59-1.12)	.22
Emergency						
No	200 (97%)	22,414 (96%)	Ref.	Ref.	Ref.	Ref.
Yes	7 (3%)	992 (4%)	0.79 (0.34-1.56)	.54	0.86 (0.36-1.74)	.71
Wound class						
1. Clean	185 (89%)	21,683 (93%)	Ref.	Ref.	Ref.	Ref.
2. Clean/contaminated	8 (4%)	574 (2%)	1.63 (0.74-3.12)	.18	1.79 (0.79-3.48)	.12
3. Contaminated	6 (3%)	789 (3%)	0.89 (0.35-1.84)	.78	0.94 (0.36-2.00)	.89
4. Dirty/infected	8 (4%)	360 (2%)	2.60 (1.17-4.98)^‡^	.009	2.13 (0.92-4.31)	.052

*OR indicates odds ratio; CI, confidence interval; and ASA, American Society of Anesthesiologists.

†All predictors were included in the multivariable model except low albumin due to few cases with an abnormal result.

‡Predictors significant at *P* < .05.

§Calculated via Fisher's Exact test, as data was too sparse for a logistic model.

‖Missing values: Readmission—ASA class = 2; Anesthesia = 1; No readmission—Gender = 3, ASA class = 267, Operation time = 1, Anesthesia = 19.

Significance is determined as *P* < .05. Those are highlighted.

**Table 2:** Postoperative adverse events associated with unplanned readmission (N = 207)

Adverse event	No	Yes
Any complication	143 (69%)	64 (31%)
Wound complication	170 (82%)	37 (18%)
Medical complication	179 (86%)	28 (14%)
Reoperation	138 (67%)	69 (33%)

**Table 3:** Procedures with the highest rates of readmission*

Procedures with the highest rate of readmission	CPT code	Procedure readmission rate	Unplanned readmission (n = 207)	No unplanned readmission (n = 23,406)	OR (95% CI), for unplanned readmission	*P*
Metacarpal—amputation, yes (n = 24)	26910	12.50%	3 (1.45%)	21 (0.09%)	16.38 (3.85- 47.97)	<.001
Finger amputation—direct, yes (n = 168)	26951	3.57%	6 (2.90%)	162 (0.69%)	4.28 (1.67-8.98)	<.001
Finger amputation—w/flap, yes (n = 184)	26952	3.26%	6 (2.90%)	178 (0.76%)	3.90 (1.52-8.15)	.001
Bennett—pinning, yes (n = 106)	26650	2.83%	3 (1.45%)	103 (0.44%)	3.33 (1.05-10.57)	.042
Tenolysis forearm, yes (n = 139)	25295	2.16%	3 (1.45%)	136 (0.58%)	2.52 (0.62-6.72)	.12
Phalanx—ORIF, yes (n = 710)	26735	1.97%	14 (6.76%)	696 (2.97%)	8.72 (4.77-14.75)	<.001
Flexor tendon—zone 2, yes (n = 425)	26356	1.88%	8 (3.86%)	417 (1.78%)	2.22 (1.00-4.23)	.029
Distal radius—comminuted, yes (n = 1430)	25609	1.61%	23 (11.11%)	1407 (6.01%)	1.95 (1.23-2.96)	.003
Distal phalanx ORIF, yes (n = 253)	26765	1.58%	4 (1.93%)	249 (1.06%)	1.83 (0.56-4.36)	.23

*CPT indicates Current Procedural Terminology; OR, odds ratio; CI, confidence interval; and ORIF, open reduction and internal fixation.

Significance is determined as *P* < .05. Those are highlighted.

**Table 4:** Comparing readmission diagnosis rates among cases with a known diagnosis (N = 93)

Reason for readmission	Number of cases	Percent of known diagnosis cases	Average days to readmission	Median days to readmission
Wound infection/disruption	39	41.94	15.9	15
Pain	13	13.98	3.46	1
Medical complications	12	12.90	12.25	13
Hardware complications	7	7.53	12.29	13
Hematoma	5	5.38	10.8	10
Tendon complications	4	4.30	16.75	16
Bleeding	2	2.15	4	4
Compartment syndrome	1	1.08	0	0
Other	10	10.75	17.8	20
